# Improving the optimized shea butter quality: a great potential of utilization for common consumers and industrials

**DOI:** 10.1186/s40064-015-1454-0

**Published:** 2015-11-04

**Authors:** Rose-Monde Megnanou, Sébastien Niamke

**Affiliations:** Laboratoire de Biotechnologies, UFR Biosciences, Université Félix Houphouët-Boigny, 22 BP 582, Abidjan 22, Côte d’Ivoire

**Keywords:** Shea butters, Optimized, Physicochemical and nutritive characteristics, Potentiality of exploitation, Industrial

## Abstract

Industrials interest in fats as raw material, resides in their exceptional quality and potentialities of exploitation in several fields. This study aimed to exalt the optimized shea butter quality and present its wide potentialities of utilization. Hence, the characteristics of beige and yellow optimized shea butters were determined. Both samples recorded very weak acid (0.280 ± 0.001 and 0.140 ± 0.001 mgKOH/g) and peroxide (0.960 ± 0.001 and 1.010 ± 0.001 mEgO_2_/kg) indexes, when the iodine indexes (52.64 ± 0.20 and 53.06 ± 0.20 gI_2_/100 g) and the unsaponifiable matters (17.61 ± 0.01 and 17.27 ± 0.01 %) were considerable. The refractive indexes (1.454 ± 0.00 and 1.453 ± 0.00) and the pH (6.50 ± 0.30 and 6.78 ± 0.30) were statistically similar; but the specific gravity (0.915 ± 0.01–0.79 ± 0.01 and 0.94 ± 0.01–0.83 ± 0.01) and the viscosity (90.41 ± 0.20–20.02 ± 0.20 and 125.37 ± 0.20–23.55 ± 0.20 MPas) differed and decreased exponentially with the temperature increasing (35–65 °C), except for the specific gravity of the yellow butter which decreased linearly. The UV–Vis spectrum showed a high peak at 300 nm and a rapid decrease from 300 to 500 nm when the near infra-red one, revealed peaks at 450, 1200, 1400, 1725 and 2150 nm for all the samples. The chromatographic profile identified palmitic (16.42 and 26.36 %), stearic (32.39 and 36.36 %), oleic (38.12 and 29.09 %), linoleic (9.72 and 5.92 %) and arachidic (1.84 and 1.59 %) acids, and also exaltolide compound (1.51 and 0.68 %). The samples also contained essential minerals (Calcium, magnesium, zinc, iron, etc.) carotene (550 ± 50 and 544 ± 50 ppm), vitamins A (0.065 ± 0.001 and 0.032 ± 0.001 µg/g) and E (2992.09 ± 1.90 and 3788.44 ± 1.90 ppm) in relatively important amounts; neither microbiological germs nor heavy were detected. All these valorizing characteristics would confer to the optimized shea butters good aptitude for exportation and exploitation in food, cosmetic and pharmaceutical industries.

## Background

Industrials interest in fats as raw material, resides in their exceptional quality and potentialities of exploitation in several fields. Hence cocoa butter, palm, palmist and coco oils are so widely exploited that the Codex Stan 210 (1999) established standards in order to guide traders/industrials. Many other fats like avocado, olive, illipe and jojoba are also involved in cosmetic or pharmaceutical products on the basis of their interesting characteristics (Pontillon 1996; Joanny 2005; AAK Global 2012) Shea (*Vitellaria paradoxa*, syn. *Butyrospermum parkii*, *B. paradoxa*) butter which represents an important socio-economical agro-resource for its producing countries, is more and more being exploited in several industrial fields (Hall et al. 1996, Schreckenberg 2004; PNUD 2010). This increasing importance is mainly linked to its properties which would justify the wide range of its empiric uses (Hall et al. 1996). Indeed, according to these authors, shea butter has long been used in sub-Saharan Africa and elsewhere for medicinal, culinary, and other applications (Tomaszkiewicz-Potepa 2012; Tomaszkiewicz-Potepa and Sliwa 2012). Nowadays, shea buter, mainly the traditional one (called BIO-shea butter) interests cosmetic and pharmaceutical firms (Pontillon 1996; Elias and Carney 2004; Nahm 2011; AAK Global 2012). Nevertheless these firms are highly exigent about shea butter quality. Whereas, the traditional processes often lead to shea butter non-conform to recommended standards (Hall et al. 1996; Mégnanou and Diopoh 2008). Several studies have been undertaken to improve shea butter properties; most of these studies just focused on one or two effecting factors. Those of Mégnanou and Niamke (2013a) took into account many factors like nuts drying mode and time, kernels quality and roasting time, so did shea butter wrapper hygienic quality. The results drew the effects of such factors on shea butter physicochemical and microbiological characteristics, and consequently induced the optimized traditional process (Bup et al. 2012; Pouliot and Elias 2013; Nsogning et al. 2014). The resulted shea butter was in conformity with the unrefined shea butter standards for trade as proposed by UEMOA (2011). Nevertheless, because of the exigencies of industrials which take into account many other properties, shea butter utilization as raw material (Cosmetic and pharmaceutical) might be linked to its exceptional quality and high potential of exploitation (Honfo et al. 2013, 2014).

The present study focused on the optimized shea butters (which process was improved) typical quality and demonstrated its high potential of exploitation in several industries. Therefore, in addition to their ordinary physicochemical characteristics like moisture content, acid, iodine, peroxide indexes and unsaponifiable matter, other characteristics have been taken into account. Hence, physical parameters such as specific gravity, viscosity and UV–Visible/infrared spectra were determined. Samples content in nutritive compound as carotene, fatty acids, minerals, vitamins A and E was also evaluated; so were the microbiological characteristics. Above all, the different aptitudes and fields of exploitation of the beige and yellow optimized shea butters have been underlined.

## Results and discussion

### Sensorial characteristics of the optimized shea butters

Both beige and yellow shea butters prepared according to the improved Megnanou et al. (2007) method, presented the same fondant (soft) texture and rancidity-less and moderate shea characteristic odor. Such characteristics are conformed to usual consumers’ criteria about shea butter (Carette et al. 2009; Mégnanou and Niamké 2013b). It is important précising about the studied samples that beige shea butter would be a natural (original) fat because of its water-exclusive extraction (Elias 2015, Jasaw et al. 2015). The yellow one, despite of the natural statute of *Cochlospermum tinctorium* dye, could be considered as an adulterate shea butter. Indeed, *Cochlospermum tinctorium* is exploited as medicinal plant; its roots aqueous extract would be drink in the treatment of many diseases (Diaw 1982). However, both beige and yellow shea butters of the present study with conform sensorial characteristics would constitute natural (BIO) available cheap and accessible edible fats. Moreover, the present optimized shea butters also exhale a slight sweety fragrance similar to that of the sweet almond oil. Such fragrance was not noticed by Megnanou et al. (2007) about the optimized shea butter, and could be justify by the improvement of the optimized method by avoiding the step of shea oil heating (for dehydration) which could destroy the volatile compounds responsible of the fragrance.

### Physicochemical characteristics of the optimized shea butters

The physicochemical characteristics presented significant difference (p < 0.05) between beige and yellow optimized shea butters, except for peroxide and refractive indexes (Table 1). Acid (0.280 ± 0.001 and 0.140 ± 0.001mgKOH/g, respectively) and peroxide (0.960 ± 0.001 and 1.01 ± 0.001mEqO_2_/kg, respectively) indexes which are considered as fat quality characteristics were very weak. Indeed, their values were at far slighter than those (4 mgKOH/g and 15 mEqO_2_/kg) recommended by Codex Stan 210 (1999) about vegetable fats. These values were also weaker than those concerning Megnanou et al. (2007) optimized shea butters shea. This situation could certainly be linked to the improved optimized method which deleted the shea oil heating step. In fact according to Dieffenbacher et al. (2000), fat heating would induce glycerids hydrolysis and unsaturated fatty acids oxidation, and consequently a high amount of free fatty acids and peroxide compounds.

**Table 1:** Physicochemical characteristics of the optimized shea butters

Parameters	Beige shea butter	Yellow shea butter
Specific gravity (40 °C)	0.87 ± 0.01b	0.92 ± 0.01a
Refractive index (40 °C)	1.454 ± 0.00a	1.453 ± 0.00a
Viscosity (mPa.s) (40 °C)	73.66 ± 0.20b	96.00 ± 0.20a
Activation energy* (kJ/mol)	46.81	50.43
Colour (Ly)	3.40 ± 0.01b	3.90 ± 0.01a
pH (25 °C)	06.50 ± 0.30b	06.78 ± 0.30a
Acid index (mgKOH/g)	0.280 ± 0.001a	0.140 ± 0.001b
Peroxide index (mEqO_2_/kg)	0.960 ± 0.001a	1.010 ± 0.001a
Iodine index (gI_2_/100 g)	52.64 ± 0.20b	53.06 ± 0.20a

Values given in table consist in means ± the standard deviation. Letters a, b resulted from ANOVA test; they must be considered line by line. Different letters (a, b) underline significant difference while the same letter (a, a) indicate no significant difference

Ea as activation energy, R as gaze constant R = 8314 × 10^−3^ kJ/mol/K. T represents the different temperatures at which viscosity was obtained; it varied from 308 to 338 °K

* Activation energy consists in the potential energy contained in one mole of shea butter. Here, it was determined graphically by drawing the function ln (Viscosity) = Ea/RT + Constante (y = Eax + constante) with

With such acid and peroxide indexes, the studied shea butter would present good aptitude for exportation/international trade though (moreover) the fat just contained 0.2 % of moisture. Additionally, values of specific gravity at 40 °C were 0.87 ± 0.00 and 0.92 ± 0.00 for the beige and the yellow shea butter, respectively. Such values added to those of the refractive index (1.454 and 1.453 for beige and yellow, respectively) and the viscosity (73.66 ± 0.20 and 96.00 ± 0.20 mPa s for beige and yellow, respectively) would confirm the quality of conventional edible vegetable oils to the shea butter samples (Codex-Alimentarius 1993; Besbes et al. 2004).

Furthermore, all studied shea butters could be classified as non-drying fats in view to their refractive index value (Rossell 1991). This property would disqualify them for varnish manufacturing in chemical industry, despite of their relatively high iodine value (52.64 ± 0.20 and 53.06 ± 0.20 gI_2_/100 g for beige and yellow shea butter, respectively) compared to that of marked shea butters reported by Megnanou et al. (2007). This iodine index value would suggest an interesting amount of unsaturated fatty acids and would confirm the very weak peroxide index.

About the viscosity, those of liquids as vegetable oil are commonly perceived as thickness, or resistance to pouring (Ndangui et al. 2010). Beige shea butter was less viscous than the yellow one, probably due to the presence of mucilage contained in *Chochlospermum tinctorium* dye (Jensen 2005). In addition, yellow shea butter viscosity was higher than the mean value (75 mPa.s) of most vegetable oils (Besbes et al. 2004). This physical property linked to the solid state of the studied shea butters could be used in food and cosmetic industry to confer an adequate texture to final fat products (Dubois et al. 2007).

**Fig. 1:**
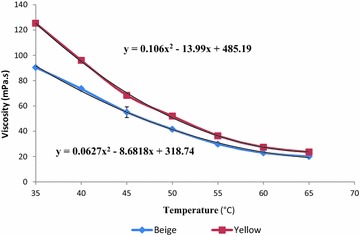
Optimized shea butters viscosity variation as function to the heating temperature. Mean values are plotted as points and standard deviations, as vertical error bars

**Fig. 2:**
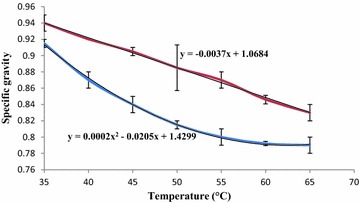
Optimized shea butters specific gravity variation as function to the heating Temperature

The profile of the temperature effect on viscosity and specific gravity are depicted on Figs. 1 and 2, respectively. Each sample observed a typical variation of viscosity (y = 0.0627x^2^ − 8.6818x + 318.74 and y = 0.106x^2^ − 13.99x + 485.19, for beige and yellow, respectively) and specific gravity (y = 0.0002x^2^ − 0.0205x + 1.4299 and y = −0.0037x + 1.0684, for beige and yellow, respectively) which were materialized by the mathematical equations on the figures. These equations (relations) could be exploited by any factory, in order to obtain precisely the viscosity/specific gravity in conformity with any utilization. In summary, the value of viscosity decreases (90.41–20.01 and 125.37–23.55 mPa.s, for beige and yellow shea butters, respectively) continuously when the temperature increases from 35 to 65 °C and would confirm the Arrhenius law (Nzikou et al. 2007). The low indicates that the viscosity of fats decreases exponentially with increasing of temperature. It was also observed a relative similitude in the evolution of the viscosity for both samples; this could suggest similarity in fatty acids composition. However, such rheological property (viscosity/temperature and specific gravity/temperature) linked to the relatively important energy of activation (46.81 and 50.43 kJ/mol for beige and yellow, respectively) of the studied shea butters could be exploited in cosmetic industry for emulsions making (Lefur and Arnaud 2004). Above all, the pH of the studied shea butters would be indicated for such industry, mainly for body care because of its values (06.50 ± 0.30 and 06.78 ± 0.30 for beige and yellow, respectively) which around the human body proteases pH (Forestier 1992).

**Fig. 3:**
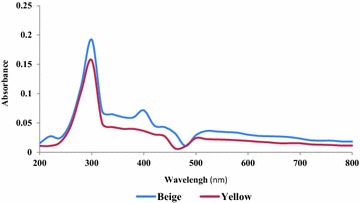
UV-Visible spectrum of optimized beige and yellow shea butters

**Fig. 4:**
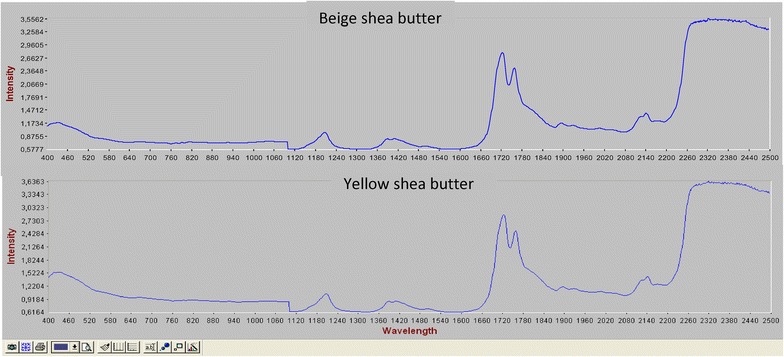
Near infrared spectrum of the optimized beige and yellow shea butters

The spectra (Near infrared and UV–visible) of both shea butters presented, at the whole, the same profile (Figs. 3, 4); which was in general similar to those of marked and original traditional shea butter reported by Mégnanou and Niamké (2014). This similitude would suggest for both beige and yellow optimized shea butters the content in molecular bearing ethylenic bonds with conjugation and carbonyl compounds (Yadav et al. 2004). The shea butters of these authors would also contain UV-filter compounds (molecular) materialized by the rapid decrease of the absorbance (0.15–0.05 and 0.2–0.075, for yellow and beige shea butters, respectively) from 300 to 400 nm (Besbes et al. 2004). Moreover, another interesting peaks were observed at 400 nm (about the beige shea butter), and at 500 nm (for both butters); they correspond to chlorophyll (A and B) and carorenoïds wavelengths, respectively. The melting of such interesting molecular would justify the great interest of cosmetic and pharmaceutical industry for fats like shea butters, and would then constitute an advantage for using the studied shea butters in cosmetic formulations as UV protectors against carcinogenic UV A and B.

The information delivered by the optimized shea butters near infrared spectra was identical to that reported by Mégnanou and Niamké (2014) about marked shea butters. Such similitude would recall and confirm the proposition of these authors to adopting the spectra (UV–visible and near infrared) as shea butter essential distinctive characteristics. Hence, any adulteration of shea butter with other fats and/or alteration would be clearly detected through the profile. As precision, the difference noticed about the intensity of peaks at 450–500 nm wavelengths, between the yellow (1.5224) and the beige (1.1734) shea butters, could be correlated to the Lovibond color value in red light (3.9 against 3.4 for the beige one). That dissimilitude would suggest the implication of carotenoids (carotene and other carotenoids) in shea butter color.

In summary, both beige and yellow optimized shea butters would contain the same compounds like hydrocarbon, unsaturated molecular, fatty acids and moleculars protecting against sun rays damages (allergy and cancer), in probably different amounts.

### Biochemical characteristics of the optimized shea butters

Biochemical characteristics also varied from a color to another, according to the Duncan’s test (Table 2). At the whole, the characteristics are very interesting and would confer to both butters a wide potential of utilization when added to the upside physicochemical characteristics. In fact, the important amount of unsaponifiable (17.61 ± 0.01 and 17.27 ± 0.01 for beige and yellow shea butters, respectively) was at far higher than those of coconut (0.2–0.4 %) and cocoa (0.5–1 %) oils currently involved in cosmetics (Karleskind 1992). It is worth noting here that the unsaponifiable fraction would contain the main potential of cosmetic and medicinal virtues (Pesquet 1992; Allal et al. 2013).

**Table 2:** Biochemical and nutritive properties of the optimized beige and yellow shea butters

Characteristics	Beige shea butter	Yellow shea butter
Unsaponifiable (%)	17.61 ± 0.01a	17.27 ± 0.01b
Vitamin A (µg/g)	0.065 ± 0.001a	0.032 ± 0.001b
Vitamin E (ppm)	2992.09 ± 1.90b	3788.44 ± 1.90a
Carotene (ppm)	550 ± 50.00a	544 ± 50.00a
Free fatty acids (%)	5.39 ± 0.05a	5.23 ± 0.05b
Ratio oleic/linoleic	3.92	4.91
SFA (%)	48.81 ± 0.09b	62.72 ± 0.09a
UFA (%)	51.19 ± 0.19a	37.28 ± 0.19b

Values given in table consist in means ± the standard deviation. Letters a, b resulted from ANOVA test; they must be considered line by line. Different letters (a, b) underline significant difference while the same letter (a, a) indicate no significant difference

In addition to their important unsaponifiable matter which would confirm their classification as “variety Mangifolia” according to Mensier (1957), the optimized shea butter contained.

carotene (550 ± 50.00 and 544 ± 50.00 ppm for beige and yellow shea butters, respectively), linoleic acid (9.72 and 5.92 % for beige and yellow shea butters, respectively), vitamins A (0.065 ± 0.001 and 0.032 ± 0.001 µg/g for beige and yellow shea butters, respectively) and E (2992.09 ± 1.90 and 3788.44 ± 1.90 ppm for beige and yellow shea butters, respectively). The presence of linoleic acid could be useful in cosmetic industry to decrease trans-epidermal water loss and to eliminate scaly lesions common in patients with essential fatty acids deficiency (Aburjai and Natsheh 2003). A natural musk fragrance identified as exaltolide was also detected in a relatively interesting amount (1.51 and 0.68 % for beige and yellow shea butters, respectively). All these characteristics would exalt their beneficial utilization in household for body care and their exploitation in cosmetic/pharmaceutical industry (body cream, soap and fragrances/perfumes). The studied samples could easily be involved in cosmetics like cocoa and jojoba fats, without preliminary treatment like deodorization, neutralization and synthetic fragrance (essence) additioning. They present fats would then constitute a valuable cheap, available and accessible material for the cosmetic/pharmaceutical industry.

**Fig. 5:**
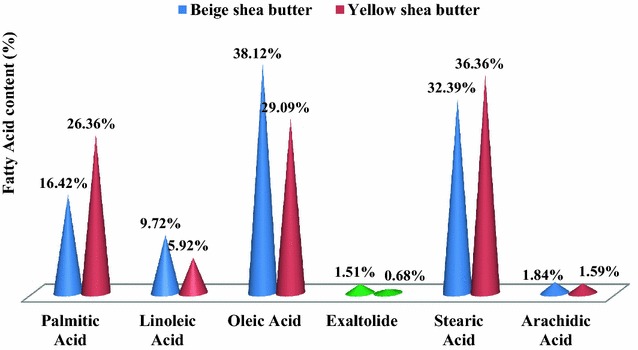
Optimized shea butters fatty acids component and content

Concerning the fatty acids composition (Fig. 5), it should underline the high nutritive potential of the optimized shea butters. Indeed, in addition to the carotene, vitamins A and E which would be powerful antioxidant, these butters contain, the fatty acids profile revealed the presence of essential fatty acids (EFA) such as oleic (38.12 and 29.09 % for beige and yellow shea butters, respectively) and linoleic (9.72 and 5.92 % for beige and yellow shea butters, respectively) acids. Their proportions were relatively important, mainly in the beige shea butter and would suggest the possibility of extracting a liquid fraction like “*olein de karite*” of Burkina Faso. Such fraction could be consumed without heating, preserving hence, vitamins (A and E), carotene and other thermo-sensible moleculars from destruction. It is worth underlining the relatively important amount of oleic acid and oleic/linoleic acids (3.92 and 4.91, for beige and yellow shea butters, respectively) ratio; such disposition would confer to the optimized shea butters the quality of very interesting dietetic fat in cardiovascular, inflammatory, autoimmune diseases and cancer prevention (Harris 2006; Simopoulos 2008). They could them be recommended for usual consumption like colza and sunflower oils which are considered nowadays as the best dietetic oil on markets. The solid fraction of the shea butters which will be constituted by stearic (32.39 and 36.36 % for beige and yellow shea butters, respectively) and palmitic (16.42 and 26.36 % for beige and yellow shea butters, respectively) acids could for it, be involved in margarine and in baking pastes to get leafy-pastes as reported by Pesquet (1992) about shea butter exploitation in bakery.

Both optimized shea butters had the same fatty acids composition, but a difference in the content was noticed and could difficultly be explained. However, the higher proportion of UFA (51.19 ± 0.19 %) recorded by the beige shea butter was in conformity with Marantz et al. (2004) repartition about west Africa shea butters. The palmitic and linoleic acids proportions were, at far more important than those of Marantz et al. (2004), despite of the absence of adulteration supposed by Mégnanou and Niamké (2014) for Ivorian marked shea butters. This situation far from constituting a scientific problem could be taken into account for a best detail in shea butter characterization as function to the country. More precisely, a correlation might be established between shea trees genetic and agro-morphological characters and their butters physicochemical and biochemical characteristics.

The carotene content was relatively weak in the yellow shea butter (544 ppm) compared with the beige one (550 ppm); this would either confirm the responsibility of *Cochlospermum tinctorium* dye in yellow butter coloration (Ayeh 1991; Megnanou et al. 2007) or and also suppose eventually the implication of vitamin E or other carotenoids (compounds different from carotene) at regard to the Near infrared observation (peaks at 450 nm).

### Microbiological and mineral characteristics of the optimized shea butters

In addition to their high physicochemical and biochemical potentialities as cosmetical/pharmaceutical and nutritive fats, the optimized shea butters contained very interesting minerals for human nutrition as calcium, magnesium, iron and copper, and for body/hair care as for zinc, magnesium and copper (Gueguen 2001). Moreover, neither microbiological germs (Table 3) nor heavy metals (Table 4) were detected. Some authors like Sanou (2002) links shea butters minerals composition to the kernels one; the present results confirmed such approach. Moreover, they suggested the integration of mineral from *Cochlospermum tinctorium* roots. Indeed, yellow shea butter composition in copper would be relative to the roots content. It is essential recalling here that the yellow optimized shea butter was prepared with the decoction of these roots (Megnanou et al. 2007).

**Table 3:** Microbiological characteristics of the optimized shea butters

Identified germs	Standard	3 × Standard	Shea butter (yellow and beige) charge
Mesophile bacteria/g	<1 × 10 + 4	<3 × 10 + 4	0
Total Coliforms/g	<25	<75	0
Yeast and moulds/g	<10	<30	0
S*almonella*/g	0	0	0

**Table 4:** Optimized shea butters mineral composition and content (mg//kg)

Samples	Copper	Iron	Lead	Nickel	Zinc	Calcium	Sodium	Magnésium	Potassium
B SB	0.95 ± 0.01	0.00	0.00	0.00	0.00	212.26 ± 0.05	84.57 ± 0.02	0.00	45.17 ± 0.05
YSB	7.15 ± 0.01	1.67 ± 0.01	0.00	0.00	1.90 ± 0.02	238.85 ± 0.05	136.62 ± 0.02	12.18 ± 0.02	36.41 ± 0.05
SK	3.30 ± 0.01	30.65 ± 0.01	0.00	0.00	9.79 ± 0.02	2151.80 ± 0.05	739.58 ± 0.05	1425.60 ± 0.05	10488.00 ± 0.05
*C.t*.r	12.95 ± 0.01	83.89 ± 0.01	0.00	0.50 ± 0.01	11.24 ± 0.02	1649.90 ± 0.05	696.32 ± 0.05	1177.95 ± 0.05	3912.75 ± 0.05

*BSB* Beige shea butter, *YSB* yellow shea butter, *SK* shea kernels, *C. t. r* *Cochlospermum tinctorium* root

The value of zero (0) germs charge recorded by the shea butters, would confirm the deep implication of shea butter conditioning in its microbiological quality (Mégnanou and Diopoh 2008; Mégnanou and Niamké 2013a).

Hence, the optimized shea butters might be apt to either exportation or industrial and households’ utilizations.

## Conclusion

The present optimized shea butters acid, moisture and peroxide values were very weak; this could be the consequence of the optimized method improvement by deleting the step of shea oil heating at the end of its preparation. Moreover, they were microbiological germs and heavy metal free. These characteristics would suggest them apt to exportation and industrial exploitation. In addition, they contained nutritional elements like essential fatty acids (oleic and linoleic acids), minerals (calcium, iron, copper, magnesium, sodium, potassium and zinc), vitamins (A and E) and carotene, which would present them as available, cheap and accessible nutritive edible fats. Such butters could then be exploited not only in households, but also in food industry, mainly edible oils factories and also in pastries/bakeries because of their high unsaponifiable matter. This latest compounds added to the presence of UV-filter molecules, vitamin E, minerals (zinc, magnesium and copper) and linoleic acid, would present them as useful raw material for cosmetic and pharmaceutical purposes. However, it should be appropriate to conserve them in hygienic conditions in order to preserve such interesting virtues. The optimized shea butters could then be considered as a response to the needs in valuable BIO-products for usual consumers for industrials. They might constitute for industrials a cheaper valuable, available and accessible raw material. Hence, adopting optimized method would guaranty the trading/exportation/industrial exploitation of this fat which is considered as the main source of money for women in shea producing area, and consequently increase monetary currency for shea producing countries.

## Methods

### Biological material

The biological material was constituted by beige and yellow optimized shea butters prepared at the laboratory following Mégnanou et al. (2007) improved method.

### Chemicals

Analytical grade solvents, standards and reagents were used to perform analysis. Solvents (n-hexane, acetic acid, diethyl-ether, ethanol, methanol and n-heptane) were provided from Merck (Germany). Standards such as fatty acids (palmitic acid, stearic acid, oleic acid, and linoleic acid) and erucic acid were purchased from Sigma-Aldrich (Germany). Wijs reagent, β-carotene, retinal palmitate and α-tocopherol were from Prolabo (France).

Microbiological analyses powders: PCA (Plate count agar), YGC (Agar with yeast, glucose *Salmonella*-*Shigella*), Mueller-Kauffmann, TS (Trypton salt), Rappaport–Vassiliadis and Kligler-Hajna were from Bio Rad. VRBL (Agar violet red blue and lactose), Hektoen and BPW (Buffered peptone water) powders provided from Diagnostic Pasteur, Scharlau Microbiology and Bio-Mérieux, respectively. The mineral analysis standard solutions (1 g/l) of calcium, copper, iron, lead, magnesium, nickel, potassium, sodium and zinc, used for mineral analysis were from Fisher.

### Methods

#### Shea butter samples preparation

Shea butters were prepared following Mégnanou et al. (2007) method improved by avoiding the step of shea oil dehydratation by heating.

Hence, Shea fruit (10 kg) pulp were revolved and the nuts were dipped in two equivalent volumes of boiling water for 20 min and then put on plates for regular sun drying for one (1) week. The nut kernels were chopped finely with a kitchen chopper and then roasted at 120–150 °C for 5 min (by part of 500 g). The roasted kernels were ground with an electric grinder (moulinex) and the paste of the kernels was boiled for 1 h in 2 equivalent volumes of distilled water for the beige shea butter and 2 equivalent volumes of a decoction of *Cochlospermum. tinctorium* roots (1 kg of roots boiled for 1 h in 10 L of distilled water), for the yellow shea butter. The floating oil of the boiling solution was delicately collected in a clean glass-pot and stores analyzed. Here, the final dehydratation by heating for 5 min was avoided.

#### Physicochemical analysis

##### Chemical indexes, specific gravity, refractive index and viscosity

Chemical indexes such as acid, peroxide and iodine indexes were obtained following AOAC (1997) methods. Specific gravity (35–65 °C) and refractive index (40 °C) of melted butters were determined following IUPAC (1979) method by using a pycnometer and a refractometer (Metller Toledo, Switzerland), respectively. Viscosity was determined at different temperatures (35–65 °C) by using a viscometer apparatus (SVM 3000, Anton Paar GmbH, Austria) equipped with a syringe filled with 1 mL of melted butter sample. Values of viscosities were automatically recorded after temperature programming.

##### pH, colour and melting point

pH value of melted butter samples was determined at 25 °C according to Afane et al. (1997) by using a pH-meter (Hanna, Hi 8915 ATC, Spain). 2 mL of melted butter sample were dissolved in 15 mL of n-hexane. The pH-meter electrode was standardized with buffer solutions (pH 4.0 and 7.0) and then, immersed into the sample to record pH value.

Colour and melting point were determined according to the MPOB (2005) methods by using a Lovibond colorimeter (Lico, Labomat, France) and a thermometric system (FP900, Metller Toledo, Switzerland), respectively.

##### UV–Visible spectra

UV–Vis spectra of melted butter samples were determined by measuring absorbance of hexanic melted butter solution (1 %) by using a UV–Visible spectrophotometer (T80+, PG Instruments, England) in the range of 200–600 nm (Besbes et al. 2004).

##### Near infrared spectrum

Near infrared spectrum (NIR) was determined by reading absorbance of melted butter sample in the range of 400–2500 nm by using an infrared spectrophotometer (Foss Liquid Analyzer, Denmark) equipped with a software (NIR Vision Spectral Analysis, Model 6500) for data acquisition.

##### Mineral detection

The mineral content of the shea butter ash was determined following the AOAC (1980) method by spectroscopy atomic absorption with a spectrophotometer *SpectrAA*-*5*. For the ash, 1 g of shea butter was mineralized in a mineralizing oven (J.P. Selecta, s.a. Ner 0346540) at 550 °C, for 24 h. The mineralization temperature increased progressively (50 °C by 30 min) from 50 to 550 °C, and then the process was stopped 24 h later.

### Biochemical and nutritive analysis

#### Unsaponifiable matter content

Unsaponifiable matter content of oil was determined following the IUPAC (1979) method. Oil sample (5 g) was saponified with 50 mL of 2 N KOH methanolic solution for 1 h. To the resulted mixture, 50 mL of distilled water was added. The unsaponifiable matter was extracted three times with 50 mL of diethyl-ether. Organic fractions were collected, washed three times with 50 mL of distilled water and then dried with sodium sulfate. Diethyl-ether was removed in a rotary evaporator (Heidolph, Hei-Vap, Germany) to recover the unsaponifiable matter which was then weighed.

#### Fatty acids composition

Gas chromatography (GC) was used to set the fatty acids profile. The compounds were first converted into their methyl esters (FAMEs) as described by the European Communities (1991) methods. Shea butter (0.1 g) was diluted in 2 mL of n-heptane and then 0.2 mL of a methanolic solution of potassium hydroxide (2 N) was added. The whole solution was shaken up for 30 s and stored for 5 min, then 1 mL of the top layer solution (FAMEs) mixed with the internal standard (erucic acid) was injected in the chromatograph (Shimadzu, GC-9A, Japan). The analysis conditions were:

- Chromatograph equipped with a mass spectrometer (MS),
- RTX5 fused silica capillary column (30 m X 0.32 mm i.d. X 0.25 µm film thickness),
- Carrier gas, helium,
- Flow rate adjusted to 23 mL/min.
- Temperatures of detector and injector, 250 °C; initial column temperature, 100 °C and programmed to increase by 5 °C per min intervals until 220 °C and, kept for 10 min at this temperature.

#### Vitamins A and E

Vitamins A and E were firstly extracted and then the different fractions were separated by high performance liquid chromatography (ACQUITY WATERS, USA). About the extraction, 200 µL of diluted shea butter (1 g of shea butter in 10 mL of hexan), were mixed with 800 µL of methanol, centrifuged and the supernatant was filtered (0.45 µm pore size). The filtered solution was used for chromatography analysis according to Gimeno et al. (2000) method under these following conditions:

- Liquid chromatography system with an optical detector TUV system
- BEH C18 column (150 × 0.25 mm i.d., 1.7 µm particle size), kept at 45 °C
- Injection volume, 10 µL
- Mobile phase, methanol/water (98/2: v/v)
- Elution flow, 2 mL/min
- Detection wavelength, 325 and 292 nm for vitamins A and E, respectively
- Vitamins A and E standards solutions were retinol palmitate and α-tocopherol acetate, respectively.

### Microbiological analysis

Microbiological analyses concerned the presence of *Salmonella* and counting microbial organisms such as aerobic mesophile bacteria (on Plate Count Agar [PCA] for 72 h), total coliforms (on Violet Red Bile Lactose agar [VRBL] at 30 °C for 24 h), thermotolerent coliforms (on VRBL at 44 °C for 24 h), yeast and moulds (on YGC at 25 °C for 72 h). The different methods used for these analyses are described by the French standards (AFNOR) numbered NF V 08-052 (AFNOR 1997), NF V 08-051 (AFNOR 1999b), NF V 08-050 (AFNOR 1999a), NF V 08-060 (AFNOR 1996) and NF V 08-059 (AFNOR 2002), respectively. For the principal suspensions, 10 g of melted shea butter were added to 90 mL of buffered peptone water.

Concerning Salmonella detection (presence), 10 g of shea butter were pre-enriched in a non-selective buffer (buffered peptone water) after incubation at 37 °C for 24 h. Aliquots of the previous solution were inoculated into selective brothes (Rappaport Vasiliadis Soy (RVS) and Salmonella-Shighella) and incubated at 42 °C and 37 °C for 24 h, respectively before being struck into *Salmonella*-*Shighella* and Hektoen Enteric agar. Both agars were incubated at 37 °C for 24 h. Several tests of confirmation were performed on typical Salmonella colonies (transparent with a black center and blue-green, respectively). Hence, after culture on Kligler-Hajna agar, urea-tryptophan and glycerol, at 37 °C for 24 h, salmonella colonies were detected by the negative results for lactose, urease, glycerol and indole, and positive result for glucose, gaz and sulfite, they presented.

### Statistical analysis

In the present experiment, each test for the sample was analyzed in triplicate. Data were expressed as mean ± standard deviation (SD). Differences between means were analysed by analysis of variance (one way ANOVA) using XLstat 2009 software. Statistical significant difference was stated at p < 0.05.
